# Neurosurgical treatment for addiction: lessons from an untold story in China and a path forward

**DOI:** 10.1093/nsr/nwz207

**Published:** 2019-12-17

**Authors:** Shuo Ma, Chencheng Zhang, Ti-fei Yuan, Douglas Steele, Valerie Voon, Bomin Sun

**Affiliations:** 1 Department of Functional Neurosurgery, Ruijin Hospital, Shanghai Jiao Tong University School of Medicine, Shanghai 200025, China; 2 Shanghai Key Laboratory of Psychotic Disorders, Shanghai Mental Health Center, Shanghai Jiao Tong University School of Medicine, Shanghai 200030, China; 3 Co-innovation Center of Neuroregeneration, Nantong University, Nantong 226001, China; 4 Division of Imaging Science and Technology, Medical School, University of Dundee, Dundee DD1 4HN, UK; 5 Department of Psychiatry, University of Cambridge, Cambridge CB2 0SZ, UK

**Keywords:** drug addiction, psychosurgery, ablative surgery, deep-brain stimulation, medical ethics

## Abstract

Addiction is a major public-health crisis associated with significant disability and mortality. Although various pharmacological and behavioral treatments are currently available, the clinical efficacy of these treatments is limited. Given this situation, there is a growing interest in finding an effective neurosurgical treatment for addiction. First, we discuss the use of ablative surgery in treating addiction. We focus on the rise and fall of nucleus accumbens ablation for addiction in China. Subsequently, we review recent studies that have explored the efficacy and safety of deep-brain-stimulation treatment for addiction. We conclude that neurosurgical procedures, particularly deep-brain stimulation, have a potentially valuable role in the management of otherwise intractable addictive disorders. Larger well-controlled clinical trials, however, are needed to assess clinical efficacy and safety. We end by discussing several key issues involved in this clinical field and identifying some areas of progress.

## INTRODUCTION

Addiction is characterized by intense and sometimes uncontrollable craving and compulsive addictive-substance seeking, which persist despite severe and potentially fatal consequences [1]. Even after prolonged abstinence, patients remain at elevated risk of relapse, especially when cues evoke memories associated with the addictive substance [2] or when patients are exposed to stress or craving symptoms [3]. Addiction-associated problems impose a high economic and social burden on society, including substantial healthcare costs due to medical complications and high-risk behaviors (e.g. needle-sharing), crime and lost work productivity [4,5]. In 2015, a global survey showed that tobacco smoking, alcohol and drug use were associated with 16.2% of the total disease burden in men [6]. In 2014, it was estimated that more than 4.9%, 3.5% and 22.5% of the world's adult population had an alcohol-use disorder, illicitly used psychoactive drugs or were addicted to tobacco products, respectively, causing an estimated 25 783 disability-adjusted years of life lost per 100 000 people. Moreover, substance-use disorders accounted for 11% of all deaths in males and for 6% of deaths in females [4]. The often chronic, relapsing nature of substance-use disorders is a key factor in contributing to their high disease burden. Today, about 85% of addicted individuals are known to relapse within 1 year of treatment, despite the availability of medications approved for relapse treatment (e.g. methadone) and various behavioral interventions developed for relapse prevention (e.g. Marlatt's intervention, self-help intervention, cue exposure therapy) [7,8]. Accordingly, the efficacy of current behavioral and pharmaceutical interventions for treating addiction is limited, which is particularly concerning given the scale of the problem. Given this situation, there is a growing interest in finding an effective neurosurgical treatment for severe and treatment-refractory cases of substance-use disorders.

**Figure 1.**
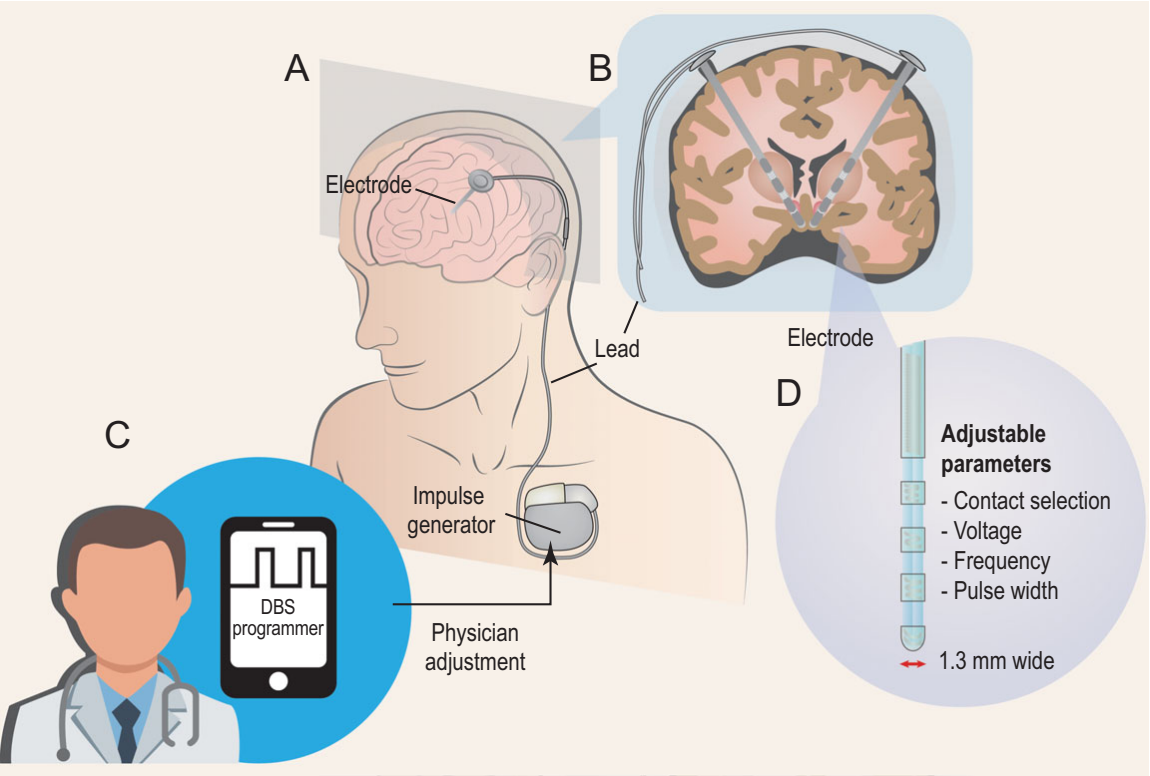
Devices for DBS and the programming system. (A) and (B) show DBS leads implanted in the brain and connected through an extension cable to a neurostimulator (impulse generator) located in the chest below the clavicle. (C) shows that a physician could program DBS using parameters including contact selection, stimulating voltage/current, frequency and pulse width (D).

Neurosurgery for treating psychiatric disorders has a long and controversial history. However, psychosurgery has regained momentum in the past few decades with the advent of deep-brain stimulation (DBS). DBS involves the delivery of electrical stimulation to gray or white matter in a therapeutic effort to change pathological brain activity (Fig. 1). The procedure is safe, effective and reversible, as well as having received US Food and Drug Administration (FDA) approvals for essential tremor since 1997 [9]. A Humanitarian Device Exemption was granted by the US FDA for DBS for dystonia in 2003 and obsessive-compulsive disorder (OCD) in 2009 [9–11]. To date, there has been mixed evidence on the efficacy of DBS for major depression, which has been attributed to patient heterogeneity, inter-individual variability and trial design [12]. More recently, several studies have also explored the efficacy and safety of DBS treatment for addiction, which we review in detail later.

**Figure 2.**
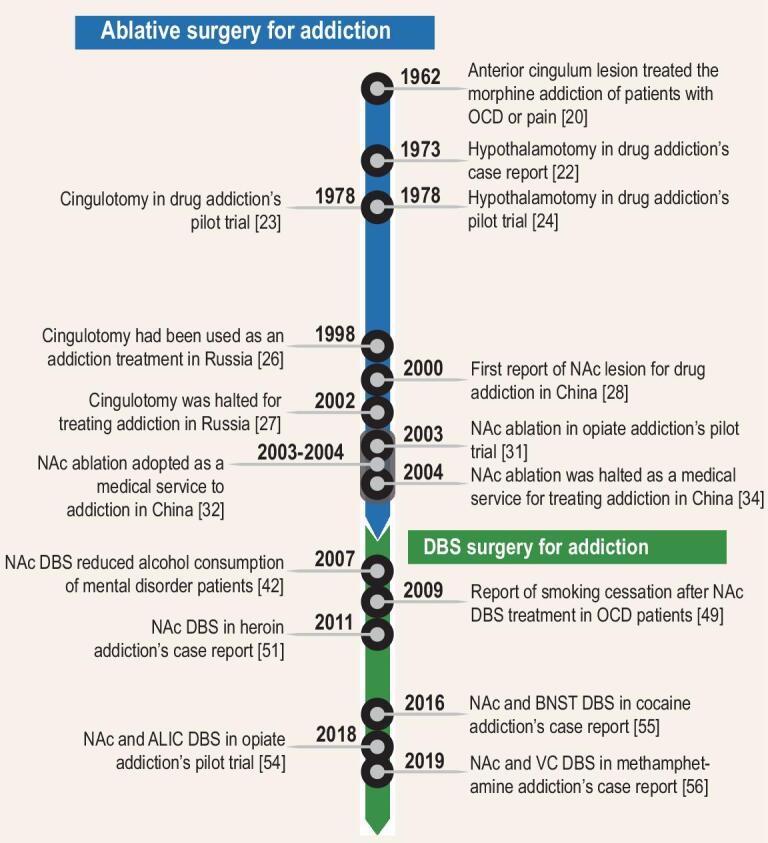
Timeline of neurosurgical-therapy development in addiction. According to the modus operandi and time sequence, neurosurgical treatment of addiction can be divided into two main stages. The first stage is represented by the ablative surgery such as bilateral cingulotomy and NAc ablative surgery whereas the second stage is represented by the DBS in various brain areas. OCD, obsessive-compulsive disorder; NAc, nucleus accumbens; DBS, deep-brain stimulation; BNST, bed nucleus of the stria terminalis.

Initially, we discuss ablative surgery, which has also been applied to addiction treatment. However, in contrast to DBS, ablative surgery is irreversible and its application to addictive disorders has provoked most debate and controversy. As an example, we focus on the rise and fall of nucleus accumbens (NAc) ablative surgery for opiate addiction in China since the early 2000s. We then review recent studies examining the utility of DBS in treating addiction (Fig. 2). Subsequently, we discuss several key issues involved in this clinical field, along with identifying some areas of progress.

## ABLATIVE SURGERY FOR ADDICTION

Since the 1920s, animal studies have disclosed an intimate structural and functional relationship between the frontal lobes and the limbic system, particularly in emotional states of fear, rage, sexual excitement, aggression and pleasure [13]. In primates, ‘loss of fear’ or ‘social indifference’ emerged following bilateral removal of the anterior cingulate gyrus [14]. Subsequently, based on the assumption that lesioning cingulate fibers might benefit mentally ill persons, cingulotomy (surgical disruption of the anatomical continuity of the cingulate gyrus) [15] was used for the treatment of various psychiatric illnesses, including mood disorders, anxiety disorders and OCD. This initial ablative-therapy study, however, yielded variable results [13,16,17]. One factor that probably has contributed to the mixed results is related to technical aspects of the neurosurgical procedure used. Although the neurosurgical procedure used targeted the cingulum bundle, it is now known that this procedure is not always precise and can also impact the adjacent cortex, which might be linked to the therapeutic effect [18,19].

In 1962, Foltz *et al.* reported the clinical outcomes of 16 patients with chronic intractable pain treated with bilateral (11/16) and unilateral (5/16) stereotactic anterior cingulum lesions. Interestingly, 14 patients who were also addicted to narcotics no longer required the drugs 72 hours after operation [16,20]. In the 1970s, based on evidence from animal studies and clinical observations, stereotactic lesions to the ventromedial hypothalamic nucleus (VMH) were also applied in the neurosurgical treatment of drug addiction [21]. One case of intractable drug and alcohol addiction was included in Müller's series of hypothalamic psychosurgery. A 30-year-old man with heavy alcohol and drug intake underwent unilateral hypothalamotomy. Despite promising early effects on his addiction and lack of adverse effects, he had to be committed to a psychiatric hospital 10 months after the surgery due to a relapse of alcoholism [21,22]. In 1978, Kanaka and Balasubramaniam analysed the clinical outcomes of 73 patients treated with cingulotomy (surgical disruption of the cingulum by mechanical, thermal or electrical lesions) for drug addiction [23]. After cingulotomy, the patients’ addictive behaviors were reduced. The improvement was particularly striking in patients addicted to meperidine, morphine or alcohol. The authors noted that the patients also showed normal psychometric test scores at follow-up, but details about these psychological measurements were not provided. The authors claimed that this procedure is a promising one for the treatment of drug addiction [23]. In the same year, Dieckmann and Schneider reported a 2- to 3-year follow-up of 13 patients treated with hypothalamotomy for their addiction to alcohol or drugs [24]. After hypothalamotomy, nine patients were able to control their consumption and the disabling aspects of addiction, such as poor social adaptation. However, significant side effects emerged. And the study concluded that bilateral hypothalamotomy is of limited value in the treatment of addiction [24].

Since 1998, bilateral cingulotomy has been used as an addiction treatment at the Institute of the Human Brain in St. Petersburg, Russia, yet with a disappointing lack of scientific documentation on patient selection, methods and clinical outcomes. The procedure involved cryosurgical lesion of the bilateral boundary between the anterior and middle thirds of the cingulate gyrus, which corresponds to Brodmann's area 24. This was accomplished through small perforations of the skull using stereotactic techniques [25,26]. In 2002, the surgical procedure was halted by the Russian authorities after a former patient complained of adverse side effects and won a court case against the institute. The patient also claimed that the operation had failed to improve his heroin addiction [26,27]. Although a subsequent report from the institute [25] appeared to suggest that bilateral cingulotomy could be effective in some addicted patients, the Russian authorities halted the surgical procedure because it was considered experimental in nature and had not been licensed by the health ministry.

In conclusion, these early ablative-therapy studies provide putative evidence that cingulumotomy, but not hypothalamotomy, could be an effective and tolerable treatment for some addicted patients. However, this conclusion should be questioned because the availability of clinical data is scarce. Well-controlled clinical trials are required to evaluate the utility and role of ablative surgery in addiction treatment.

## ABLATIVE SURGERY FOR DRUG ADDICTION IN CHINA

### Early days

In 2000, a hospital in Xi'an launched the first NAc ablative surgery for treating drug addiction in China [28]. The treatment offered was based on preclinical studies using a conditioned place-preference model of drug-seeking behavior in rats and rhesus monkeys [28–30]. The results demonstrated that electrolytic lesions of the NAc markedly decreased morphine-seeking behavior. The researchers postulated that the NAc, as compared to other basal ganglia nuclei, is the most important component of the brain's mesolimbic system involved in drug reinforcement and addiction. Based on the preclinical results, the authors claimed that NAc ablation could be an effective neurosurgical treatment option for opiate addiction in humans [28].

In 2003, Gao *et al.* reported the clinical outcomes of 28 patients treated with NAc ablation for opiate addiction [31]. After NAc ablation, the patient outcomes were encouraging, showing relapse rates of 8% (within 1 month), 39% (between 1 and 6 months) and 58% (after more than 6 months). As for the reported side effects, four patients experienced temporary memory loss and two patients had personality changes. However, these side effects apparently did not affect the patients’ daily functioning or intellectual ability [31].

### Ablative surgery becomes popular in China and issues emerge

The study of Gao *et al.* published in 2003 attracted wide attention in the neurosurgery field. The reported clinical benefits of NAc ablation to the opiate-addicted patients were generally modest and progressively diminished over the follow-ups. The results of NAc ablative surgery in treating addiction still encouraged the neurosurgeons in psychosurgery and brought hope for patients who suffered from intractable drug addiction. However, some private hospitals driven by financial interests used this procedure as a medical service mostly for economic profit. Conceivably, some patients believed that, finally and fortunately, an effective, easy, novel treatment for drug addiction was available. Ablative surgery became rapidly and widely adopted as a suitable treatment approach to opiate addiction in China between 2003 and 2004, despite the lack of solid clinical data. In December 2004, at least 1000 registered patients had received ablative surgery for opiate addiction [28,32].

Very shortly, several patients reported suffering from severe side effects after the surgical treatment, which attracted extensive media and public attention [33]. For example, journalists from China Central Television interviewed 50 patients who had received NAc ablative surgery in a specialized hospital in Guangdong province in November 2004. The patients were interviewed between 2 and 8 months after their ablative surgery. Six patients had experienced a relapse, but the other 44 patients were still abstinent. However, 26 of the 44 abstinent patients developed various significant adverse effects. It should be emphasized, however, that 42 out of the 44 abstinent patients had also used naltrexone as an adjuvant therapy after their surgery. This makes it difficult to infer whether the clinical improvements and side effects seen in these patients were mediated by the ablative therapy, the adjuvant therapy or by both treatments combined [33].

### Moratorium declared on ablative surgery

The rapid and widespread adoption of NAc ablative surgery for drug addiction sparked widespread debate and controversy in China. Many medical experts questioned the effectiveness and safety of the neurosurgical procedure. The further controversy involved the issue of whether patients should pay for experimental surgery. In November 2004, China's Ministry of Health published the results of the Symposium on Clinical Research on Clinical Detoxification of the Ministry of Health, conveying three important points. First, NAc ablative surgery for addiction was halted as a medical service. Second, neurosurgical treatment (including NAc ablative surgery) for addiction was considered a potentially promising means to prevent drug relapse, warranting appropriate clinical studies on efficacy and safety under strict management and supervision. Third, clinical studies on the long-term outcomes of patients who had received NAc ablative surgery were required [34].

### Lessons to be learned

This historical perspective highlights that experimental ablative surgery for addiction was too rapidly incorporated into routine clinical care in China. The most important things that we should ascertain are the reasons for the widespread rise and fall of ablative surgery for addiction. First, from a scientific point of view, well-controlled clinical trials had not been conducted to support the claim that NAc ablative surgery was a safe and effective treatment for drug addiction in humans [35–37]. Thus, there existed no solid scientific basis for its widespread clinical use. Meanwhile, China had a large population of drug abusers (i.e. the cumulative number of registered drug users in China increased to 1.05 million in 2003) [38] and they lacked effective measures to curb drug relapse. This represented a huge market for ablative surgery in treating drug addiction. Often driven by financial interests, some individuals and private hospitals increased their advertising and directly performed NAc ablative surgery as a medical service for addicts. Some private hospitals even performed the experimental surgery without enough expertise in stereotactic procedures. These medical malpractices inevitably damaged some patients’ health, while also restricting the development of psychosurgery in the long run.

From an ethical point of view, ablative surgery is an irreversible intervention. The neurosurgeons can only operate the surgery with patients’ informed consent. Although the surgical procedures performed in hospitals across China were approved by their own local ethical committees, the neurosurgical treatment for drug addiction had not received official general approval. Hence, the application of ablative surgery to addiction treatment in clinical practice was premature, irresponsible and therefore halted by China's Ministry of Health. In China, almost every hospital has its own ethics committee including hospital directors, medical experts and full-time managerial staff. In practice, the ethics committees in some hospitals represent just a formality. Therefore, it also became clear that the functioning of local hospitals’ ethics committees should be improved to ensure that basic scientific and ethical standards are strictly followed in human research and clinical practice [35,39].

## DBS SURGERY FOR ADDICTION

In addition to ablative surgery, DBS has been used to treat drug addiction. A comprehensive search of the PubMed database for DBS studies was performed on 25 March 2019. We consulted the Institute of Medicine's Standards for Systematic Reviews, as well as the PRISMA Group guidelines [40,41] in establishing the study-eligibility criteria. First, we conducted a search using the Medical Subject Heading (MeSH) Terms ‘deep brain stimulation’ and ‘addiction, substance’, which yielded 87 search results. Next, this number was reduced to 58 after applying the following three study-eligibility criteria: the study involved human subjects, the study results were published between 1 January 1987 through 1 March 2019 and the study report was written in English or Chinese language. Only clinical studies of patients with psychiatric and/or substance-use disorders were of interest for this review. Studies were eliminated that involved patients with neurologic disorders (e.g. Parkinson's disease) or employed clinical-outcome measures other than a substance-use-related outcome. The reference lists of eligible study papers were reviewed to identify additional eligible papers. Finally, the total number of eligible papers identified by the stepwise search process was 17 (Table 1). The included DBS-treatment studies focused on alcohol (*n* = 7), tobacco smoking (*n* = 2), heroin (*n* = 5), cocaine (*n* = 1) and methamphetamine (MA) (*n* = 2).

**Table 1.** Chronological listing of DBS treatment for addiction studies included in the review

Studies	*n*	Participants	Follow-up period	DBS targets	DBS parameters	Clinical effects	Adverse effects
Kuhn *et al.* (2007) [42]	1	Alcohol-dependent; intractable agoraphobia	1 year	Bilateral NAc	90 μs, 130 Hz, 3–4.5 V	Reduction of alcohol use	No side effects
Müller *et al.* (2009) [43]	3	Alcohol-dependent	1 year	Bilateral NAc	90 μs, 130 Hz, 3.5/4.5 V	Two participants maintained abstinence, while the other relapsed	Hypomania
Kuhn *et al.* (2011) [44]	1	Alcohol-dependent	1 year	Bilateral NAc	120 μs, 130 Hz, 5.5 V	Reduction in alcohol use	NS
Müller *et al.* (2016) [45]	5	Alcohol-dependent	4–8 years	Bilateral NAc	90 μs, 130 Hz, 3.5/4.5 V	Two patients maintained abstinence for >7 years and the others relapsed	Hypomania
Voges *et al.* (2013) [46]	5	Alcohol-dependent	31–47 months	Bilateral NAc	90 μs, 130 Hz, 4.5 V	Two participants maintained abstinence for >4 years, one showed a reduction in alcohol use and two relapsed	Hypomania
De Ridder *et al.* (2016) [47]	1	Alcohol-dependent	18 months	Bilateral dorsal anterior cingulate/supplementary motor area	1000 μs, 3 Hz, 1.5 mA	The participant maintained abstinence for >18 months	NS
De Ridder *et al.* (2017) [48]	1	Alcohol-dependent; refractory OCD; anxiety; depression	9 months	Bilateral dorsal anterior cingulate cortex	3-Hz burst mode	Modest reduction in alcohol use	NS
Kuhn *et al.* (2009) [49]	10	Smokers with refractory Tourette's syndrome, OCD or anxiety disorders	30 months	Unilateral/bilateral NAc	90/180 μs, 130/140/145 Hz, 3–6.5 V	A higher rate of successful smoking cessation than general population	NS
Mantione *et al.* (2010) [50]	1	Smokers with refractory OCD and obesity	2 years	Bilateral NAc	90 μs, 185 Hz, 3.5 V	The participant lost weight and stopped smoking	NS
Zhou *et al.* (2011) [51]	1	Heroin-dependent	6 years	Bilateral NAc	90 μs, 145 Hz, 0.8–2.5 V	The participant stopped drug abuse completely	Mild confusion; urine incontinence
Valencia-Alfonso *et al.* (2012) [52]	1	Heroin-dependent	6 months	Bilateral borders of the internal capsule and nucleus accumbens	90 μs, 180 Hz, 3.5 V	The participant remained drug-free for >6 months (except for one relapse)	NS
Kuhn *et al.* (2014) [53]	2	Heroin-dependent; various drug abuse	1/2 years	Bilateral NAc	90/120 μs, 130/140 Hz, 4.5/5.0 V	Patients remained off heroin (except for one relapse)	Epileptic seizure
Chen *et al.* (2018) [54]	8	Heroin-dependent	2 years	Bilateral NAc and the neighboring anterior limb of the internal capsule	150–240 μs, 130–185 Hz, 1.5–7.0 V	Five participants remained abstinent for >3 years and two relapsed	Intracranial hemorrhage; memory decline; dizziness; agitation/irritability; sweating; difficulty in falling asleep
Zhang *et al.* (2018) [60]	1	Heroin-dependent; hepatitis C; syphilis; antisocial personality disorder	3 months	Bilateral ventral capsule/ventral striatum	90 μs, 130 Hz, 2.5–5.5 V	The participant died from a heroin overdose	/
Goncalves-Ferreira *et al.* (2016) [55]	1	Cocaine-dependent; various drug abuse	2.5 years	Bilateral posterior-medial part of NAc and neighboring BNSTs	150 μs, 130/150 Hz, 2.0–4.0 V	Reduction in cocaine use	Warmness; sweating; flushing; occasional metallic taste; transient weight gain; diminished libido
Zhang *et al.* (2019) [56]	1	MA-dependent	1 year	Bilateral NAc and ventral capsule	90 μs, 130 Hz, 2.5 V	The participant remained drug-free and his social functions were improved	No side effects
Ge *et al.* (2019) [57]	2	MA-dependent	1.5/2.5 years	Bilateral NAc and the neighboring anterior limb of the internal capsule	210/240 μs, 150/165 Hz, 2.5/3.3 V	One participant remained abstinent, while the other relapsed	Insomnia; teeth grinding; hypomania

DBS, deep-brain stimulation; OCD, obsessive-compulsive disorder; STN, subthalamic nucleus; MA, methamphetamine; NAc, nucleus accumbens; NS, not specified.

### Clinical findings

#### Alcohol

Kuhn *et al.* observed that a patient consumed remarkably less alcohol while being treated with NAc DBS for refractory agoraphobia with panic attacks along with depression [42]. His anxiety disorder and depression, however, were little or not affected following the neurosurgical treatment. A pilot study of Müller *et al.* substantiated this initial clinical observation [43]. Additionally, Kuhn *et al.* described a 69-year-old male patient with chronic alcohol abuse who stopped drinking alcohol all together after 1 year of bilateral NAc DBS [44]. Subsequently, Müller *et al.* reported the long-term (up to 8 years’) outcomes of five patients treated with NAc DBS for alcohol addiction. During treatment, two patients remained abstinent for more than 7 years, while three patients substantially reduced their alcohol consumption [45]. Also, Voges *et al.* reported that five alcohol-addicted patients were successfully treated (average follow-up 38 months) with NAc DBS [46]. Additionally, De Ridder *et al.* presented data from two patients indicating that the anterior cingulate cortex could also be an effective DBS target in treatment for alcohol addiction [47,48].

#### Tobacco smoking

A decade ago, Kuhn *et al.* described 10 patients who received NAc DBS treatment for Tourette's syndrome, OCD or an anxiety disorder [49]. All patients were also tobacco smokers before treatment. During 1-, 2- and 2.5-year follow-ups, the researchers incidentally observed a higher rate of successful smoking cessation among the patients (20%, 30% and 30%) when compared to their reported rate of unaided smoking cessation in the general population (13%, 19% and 9%) [49]. Similarly, Mantione *et al.* incidentally observed the possible effectiveness of NAc DBS in modifying tobacco-smoking behavior [50].

#### Heroin

To our knowledge, Zhou *et al.* were the first to describe a 24-year-old male patient treated successfully with NAc DBS for heroin addiction. In the 6-year follow-up, the patient remained relapse-free, as confirmed by laboratory tests, as well as displaying improved cognitive function and reduced co-morbid symptoms of anxiety and depression. Immediately after surgery, the patient experienced mild confusion and urine incontinence, which resolved within 12 hours. No other significant side effects or complications were reported [51].

Valencia-Alfonso *et al.* similarly described a heroin-addicted male patient treated successfully with DBS of the NAc and adjacent internal capsule. After DBS surgery, the patient's heroin use and craving decreased progressively over the first 4 months of treatment. Subsequently, he was able to stop using heroin all together. At the final follow-up, the patient was drug-free for more than 6 months, except for a 14-day relapse [52].

In line with these case reports, Kuhn *et al.* reported that NAc DBS was effective in treating two patients who were chronic users of heroin as well as of other addictive drugs. After treatment, both patients showed also reduced levels of anxiety and depression [53]. Furthermore, Chen *et al.* described the outcomes of eight patients who had received DBS of the NAc and anterior limb of the internal capsule for addiction to heroin and other drugs [54]. With chronic DBS, five patients were abstinent for more than 3 years, two relapsed after abstinence for 6 months and one was lost to follow-up. The authors reported the occurrence of adverse events, including an intracranial hemorrhage (<3 ml) adjacent to the implanted electrode in one patient and a slight memory decline during chronic stimulation in another patient.

#### Cocaine

Goncalves-Ferreira *et al.* [55] described a patient who was treated with DBS of the NAc and the neighboring bed nucleus of the stria terminalis for cocaine addiction. Six months after continuous DBS, the patient's cocaine intake and craving were markedly reduced. Two years after DBS, the clinical benefits were still evident, yet they were smaller than

 

observed at 6-month follow-up. Side effects of the treatment were not lasting and rapidly resolved by adjusting the DBS parameters.

#### MA

Zhang *et al.* described a patient with a 5-year history of intractable MA-use disorder who presented with no other co-morbid psychiatric or substance-use disorders [56]. During the 1 year of DBS of the NAc and ventral capsule, the patient remained drug-free and his social functioning greatly improved. No significant side effects were reported. Interestingly, the patient also underwent positron emission tomography (PET-CT) and displayed a marked increase in striatal DAT density at the 1-year follow-up (20.5% increase in the caudate, 25.6% increase in the putamen relative to 3.2% change in the frontal cortex), paralleling the clinical benefits of the DBS treatment [56]. Additionally, Ge *et al.* reported on the outcomes of two cases of MA addiction treated with bilateral NAc DBS [57]. During the approximately 2-year follow-up period, one patient remained MA-abstinent and reported experiencing more pleasant emotions. By comparison, the other patient showed no clinical response to NAc DBS and subsequently relapsed. According to the authors, a plausible explanation for the discrepancy between the two patients’ clinical outcomes could be related to the spatial accuracy and location stability of the implanted DBS electrodes. Treatment side effects reported included insomnia, teeth grinding and a hypomanic period for less than 1 week, which remitted after adaptation of the stimulation parameters [57].

The above clinical data suggest that DBS could have a valuable role to play in the clinical management of patients addicted to various psychoactive substances, including alcohol, tobacco smoking, opiates, cocaine and MA. Also, DBS has been found to reduce impulsive and compulsive behaviors, including abuse of dopaminergic medications and pathological gambling, associated with Parkinson's disease [58,59]. However, the currently available data on

DBS treatment for addiction are extremely limited and the evidence has come primarily from case reports and case series and shows uneven results, with several subjects reducing and a minority even stopping their addictive behaviors (for details of DBS-treatment effects on all the 39 patients, see Supplementary Materials). Meanwhile, various side effects of DBS addiction treatment have been documented, such as dizziness, agitation and insomnia, but the side effects reported are usually transient, not severe and remitted by adjusting the stimulation parameters. It has been noted that a male patient with a long history of drug abuse died from a heroin overdose after 3 months of NAc DBS treatment [60]. It is unknown, however, whether the fatal outcome of this patient bears any direct relation to the DBS treatment that he received.

## FUTURE DIRECTIONS IN DBS TREATMENT FOR ADDICTION

**Figure 3.**
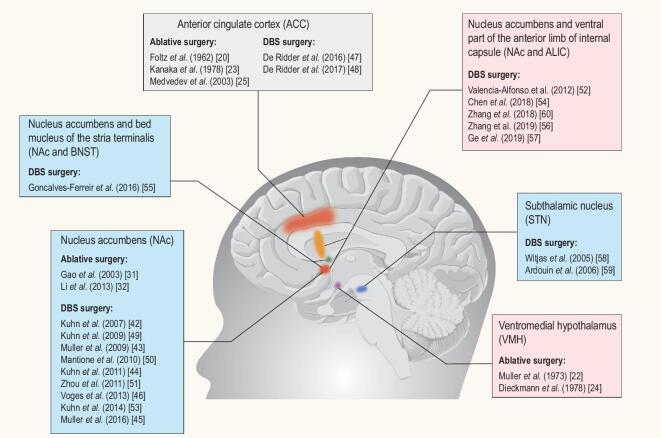
DBS and ablative-surgery targets in the management of addiction. The targets that have been used for neurosurgical treatment for addiction mainly include: ACC, NAc, STN, NAc and the neighboring ALIC, NAc and the neighboring BNST, and VMH. Among them, the ACC and NAc targets have been both used for DBS and ablative surgery. The STN, NAc and the neighboring ALIC, NAc and the neighboring BNST targets were only used for DBS surgery.

Several areas of progress in DBS treatment for addiction may be identified. First, as alluded to earlier, randomized–controlled studies are urgently needed to evaluate the efficacy and safety of DBS treatment for addiction. Also, uncertainty still exists about the best target and stimulation parameters for DBS addiction treatment. Although the NAc seems to be one of the most relevant and widely used targets, DBS of the NAc combined with the ventral internal capsule or DBS of other mechanistically informed targets, such as the subthalamic nucleus, lateral habenula, medial forebrain bundle or bed nucleus of the stria terminalis, could also be valuable targets (Fig. 3). In fact, even with the use of NAc or ventral internal capsule targets, it remains unclear which white-matter bundles (e.g. dopaminergic medial forebrain bundle, dorsal versus ventral anterior thalamic tracts or amygdalofugal) are associated with the greatest clinical benefits to the patients. In addition, it is unknown whether different targets and stimulation parameters may be more effective for patients who are addicted to a certain kind of substance.

DBS seems to be effective for some, but not all, addicts. The bases of these inter-individual differences in clinical response and outcome remain to be elucidated. Consequently, studies are needed to examine the potential role of demographics (e.g. age, gender, socio-economic status, ethnicity) and clinical features (e.g. age of onset of drug use, severity and chronicity of drug use, multiple substance use, co-morbid psychiatric disorders, family history of psychiatric and/or substance-use disorders) in modifying patient outcomes. Also, whether the patient has a supporting social network available could also play a significant role in the recovery. The problem of clinical heterogeneity, however, is related to or compounded by the likelihood that patients also differ from each other in terms of etiology—that is, in the specific genetic [61], epigenetic [62] and environmental risk factors [63–65] that are involved in their addictive behaviors. Finally, neuroanatomic differences between individual patients, affecting the organization of fiber tracts, are possibly another source of patient-efficacy variability. It seems, therefore, that patients clinically diagnosed with a certain substance-use disorder form a genetically, etiologically and clinically heterogeneous patient population, which makes it difficult to produce study results that are identical or comparable across patients and studies. Ultimately, an individualized therapeutic strategy based on multimodal approaches is crucial to optimize the efficacy and tolerability of DBS treatment for addiction.

Non-invasive neuromodulation techniques, such as repetitive transcranial magnetic stimulation (rTMS) and transcranial direct current stimulation (tDCS), have also provided clinical data on their effects on craving, cue reactivity, use and relapse in addictive disorders, which have been summarized by a recent review [66]. It is clear that each neuromodulation technique has practical advantages and disadvantages. For example, many brain regions implicated in addiction, such as the NAc and subthalamic nucleus, cannot be targeted using tDCS or rTMS. In practice, the use of multimodal neuromodulation techniques should be taken into account. One could argue that non-invasive stimulation therapies like rTMS or tDCS could be initially attempted and, in case there is no long-lasting response, DBS could be a last resort to treat addiction in refractory patients [67]. De Ridder and colleagues applied this approach to target the dorsal anterior cingulate cortex (dACC) for rTMS based on functional magnetic resonance imaging (fMRI) and source-localized resting-state electroencephalograph. The rTMS treatment exerted a short-lasting (6 weeks’) clinically meaningful improvement. For ongoing stimulation, two ‘back-to-back’ paddle electrodes were implanted bilaterally in the dACC. After DBS, the patient remained free of alcohol intake and experienced reduced levels of agoraphobia over the 18-month follow-up [47]. In addition to fMRI, which has been used for exploring effective DBS targets [47], other neuroimaging techniques, such as PET-CT, have also been used for exploring the mechanism of DBS treatment for addiction [56]. However, reproducibility in neuroimaging is hindered by the fact that neuroimaging data are typically derived from group-level analyses and may not come true at the level of an individual [68]. The use of supervised machine learning provides more information on the whole-brain neural correlates of addictive processes that may be applied at the individual level. More importantly, well-controlled prospective studies are needed to ascertain whether insights from neuroimaging are able to provide reliable brain biomarkers for addiction treatment [68].

Given rapid dynamic change related to the key neurocognitive processes associated with addiction, high-temporal-resolution signals of human-brain processing based on electrophysiology, namely event-related potentials (ERPs) may enrich our understanding of the neural mechanisms of addiction from a more dynamic and comprehensive perspective. For instance, Valencia-Alfonso and colleagues’ work showed us that pretreatment recordings of the implanted target in response to symptom triggers can help to determine the clinically most effective target for DBS stimulation and facilitate custom-tailored DBS treatment [52]. Meanwhile, a diverse range of ERPs can act as the index of behavior change in patients with addiction [69]. However, few of the addictive-behavior-associated ERPs have been used for monitoring the therapeutic effect of DBS treatment for addiction.

A promising new technique towards a person-oriented approach to DBS treatment is known as a ‘closed-loop’ DBS system. This technique refers to a closed-loop feedback control system that can detect certain biological signals in the brain, such as beta local field potential levels in patients with Parkinson's disease. Subsequently, if therapeutically required, the system can use the biological signal of interest to change the DBS-treatment parameters and thereby titrate the detected signal to a desired range. Closed-loop DBS has already been approved by the US FDA for epilepsy treatment [70]. Unfortunately, closed-loop DBS is not yet available for addiction treatment because of the absence of reports on effective and feasible biological signals or biomarkers that are both sensitive and specific to the mental and brain state of interest (e.g. craving, cue- or stress-induced intentions, or impulses to seek and take drugs) and suitable for clinical use. When such biomarkers become available, DBS for addiction treatment could become individually tuned and more focused on the pathophysiological process involved, ultimately making it more clinically effective [66,71].

## CONCLUSION

In the past half-century, neurosurgeons have endeavored to find effective and safe operations for the treatment of addiction. However, due to the lack of high-level clinical trials and proper scientific understanding for these procedures, there is limited evidence in favor of any operation for the treatment of addiction to date. With the advance of technical approaches and understanding of neurophysiology, as well as the accumulation of high-level clinical trials’ data, the surgical treatment for addiction will continue to move towards a safer and more standardized direction.

## Supplementary Material

nwz207_Supplemental_File
